# Functional connectivity of the entorhinal–hippocampal space circuit

**DOI:** 10.1098/rstb.2012.0516

**Published:** 2014-02-05

**Authors:** Sheng-Jia Zhang, Jing Ye, Jonathan J. Couey, Menno Witter, Edvard I. Moser, May-Britt Moser

**Affiliations:** 1 Centre for Neural Computation and Kavli Institute for Systems Neuroscience, Norwegian University of Science and Technology, 7489 Trondheim, Norway; 2 Department of Psychiatry, Erasmus Medical Center, Rotterdam, The Netherlands

**Keywords:** hippocampus, entorhinal cortex, grid cells, border cells, place cells, optogenetics

## Abstract

The mammalian space circuit is known to contain several functionally specialized cell types, such as place cells in the hippocampus and grid cells, head-direction cells and border cells in the medial entorhinal cortex (MEC). The interaction between the entorhinal and hippocampal spatial representations is poorly understood, however. We have developed an optogenetic strategy to identify functionally defined cell types in the MEC that project directly to the hippocampus. By expressing channelrhodopsin-2 (ChR2) selectively in the hippocampus-projecting subset of entorhinal projection neurons, we were able to use light-evoked discharge as an instrument to determine whether specific entorhinal cell groups—such as grid cells, border cells and head-direction cells—have direct hippocampal projections. Photoinduced firing was observed at fixed minimal latencies in all functional cell categories, with grid cells as the most abundant hippocampus-projecting spatial cell type. We discuss how photoexcitation experiments can be used to distinguish the subset of hippocampus-projecting entorhinal neurons from neurons that are activated indirectly through the network. The functional breadth of entorhinal input implied by this analysis opens up the potential for rich dynamic interactions between place cells in the hippocampus and different functional cell types in the entorhinal cortex (EC).

## 1. Introduction

The hippocampal and parahippocampal cortices contain several cell types with distinct spatial firing patterns. The first cell type to be discovered was the place cell, which in small environments typically fires repeatedly and selectively in a single region of the available space, the cell's place field [1,2]. In larger environments, hippocampal cells can have more than one place field, without any striking spatial arrangement of the firing fields [3,4]. The fact that the majority of hippocampal principal cells are place cells [5–9] suggested early on that location is a major component of the functional output of the hippocampus [2], but it remained unclear how the space signal was generated. It was clear that the pattern was not merely extracted from sensory inputs, but the mechanism that generated the space signal, and its location in the brain, were not apparent.

**Figure 1.**
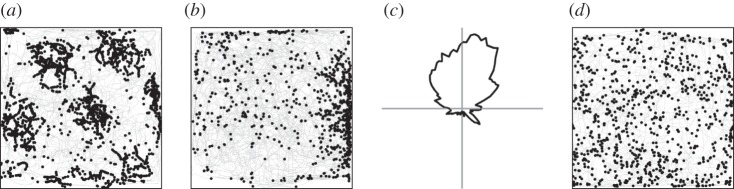
(*a*) Grid cell, (*b*) border cell, (*c*) head-direction cell and (*d*) non-spatial cell. For the grid cell, the border cell and the non-spatial cell, the animal's trajectory is shown in grey with spike locations superimposed in black. For the head-direction cell, firing rate is shown as a function of head-direction.

Following the discovery of place cells, accumulating evidence raised the possibility that the hippocampal place signal was not generated within the hippocampus itself [10,11]. An obvious place to look for an external origin was the entorhinal cortex (EC). The EC provides nearly all of the cortical input to the hippocampus, with the exception of weak components from the pre- and parasubiculum to the dentate gyrus [12,13], and from postrhinal cortex to CA1 [14], and a more substantial input from perirhinal cortex to CA1 [15]. The majority of the entorhinal projections to the dorsal hippocampus, where place cells have easily identifiable firing fields in small standard-sized recording environments, come from the dorsal part of the medial entorhinal cortex (MEC), near the postrhinal and perirhinal cortices [16,17]. When activity was recorded from this part of the EC for the first time in the early 2000s, it turned out that a large fraction of the principal neurons in the MEC had discrete firing fields, reminiscent of the place fields of hippocampal neurons [18]. Subsequent work showed that there are several types of cells with spatial firing correlates in this area. The predominant cell type is the grid cell (figure 1*a*), whose multiple firing fields form a periodic hexagonal lattice covering the entire space available to the animal [19]. The network of grid cells is organized in a modular manner, with cells clustering into semi-topographically arranged subgroups with similar grid spacing and grid orientation [20]. Grid cells co-localize with head-direction cells, which fire only when the animal faces a certain direction [21–23], and border cells, which fire specifically when the animal is near one or several borders of the local environment [24,25] (figure 1*b*). It is likely that place cells are created by output from one or several of these entorhinal cell types, although it has not been determined which cell types contribute to this process, given that only a subset of the entorhinal projection neurons target the hippocampus [26].

The conversion from spatial signals in the MEC to place fields in the hippocampus, only one synapse downstream, is probably one of the most accessible examples of a neural-code transformation in the higher mammalian cortices. In this paper, we review recent work from our laboratory in which we have tried to identify the spatial cell types of the MEC that project directly to the hippocampus and that may provide the spatial input from which place fields are generated [27]. With the origin of place fields as a guiding question, we demonstrate how optogenetics can be used to distinguish cells with direct hippocampal projections from cells that target hippocampal neurons only indirectly via other cells. The implications of findings from recent experiments using these methods will be discussed and related to models of place-cell formation.

## 2. Functional identity of hippocampus-projecting medial entorhinal cortex cells

A classical method for determining whether a recorded neuron projects to a given brain location involves antidromic stimulation. Axons in the putative projection area are stimulated at the same time as spikes are recorded from the putative parent cell. If the stimulation is followed by short-latency discharge in the recorded cell, and if discharge can be blocked by appropriately timed oppositely directed spikes from the soma, then it is safe to conclude that the cell sends axons to the target area. However, a major disadvantage of this strategy is that only axons near the tip of the electrode are activated. In the hippocampus, for example, a single electrode can recruit only a small fraction of the entorhinal projection neurons, even when the electrode is placed in the centre of the fibre bundle and stimulation intensities are maximized [28]. Negative results with antidromic stimulation are therefore hard to interpret as the recorded cell may send axons to the target region (the hippocampus) that bypass the tip of the stimulation electrode.

**Figure 2.**
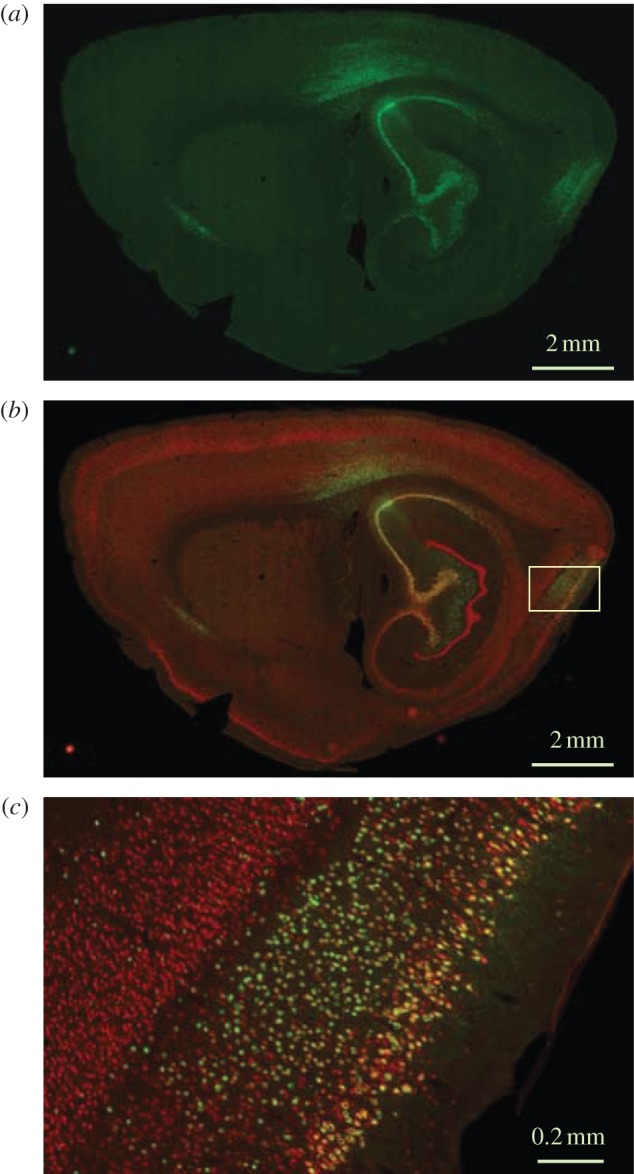
Sagittal sections showing viral transduction of hippocampus-projecting neurons in MEC. EYFP-carrying rAAV was injected in the dorsal hippocampus. (*a*) EYFP expression at low magnification (green), sagittal section; (*b*) section co-stained for NeuN (red), low magnification; (*c*) high magnification of the framed area in (*b*). The framed area shows the dorsal part of MEC. Note co-expression of EYFP and NeuN in layer II–III but not layer V–VI cells of the MEC. Modified with permission from [27].

In response to this challenge, we have recently developed a method for functional tagging of neurons with axonal projections to a region of interest in which we bypass the limited spatial range of conventional stimulation electrodes [27]. A viral vector was used to induce expression of a light-responsive transgene in the subset of MEC neurons that project directly to the hippocampus. The viral vector was a recombinant adeno-associated virus (rAAV) carrying genes for the light-sensitive cation channel channelrhodopsin-2 (ChR2) as well as a marker protein such as EYFP or FLAG. The virus was infused into the dorsal hippocampus, where it transduced local cells as well as axons from neurons with cell bodies in other brain regions, such as the EC. Retrograde transport of virus from axons to soma was maximized by cross-packaging rAAV2 with the viral capsid of AAV1 to generate chimeric rAAV2/1. Membrane trafficking was improved by a trafficking signal, and an endoplasmic reticulum exporting motif derived from the inward-rectifier potassium ion channel Kir2.1 [29]. Rats injected with EYFP-carrying rAAV2/1 in the hippocampus showed strong EYFP expression not only at the injection site but also in the layers II and III of the EC (figure 2). Only minimal staining was observed in the deep layers of EC, as expected in this area if only neurons with direct projections to the hippocampal injection site were infected.

**Figure 3.**
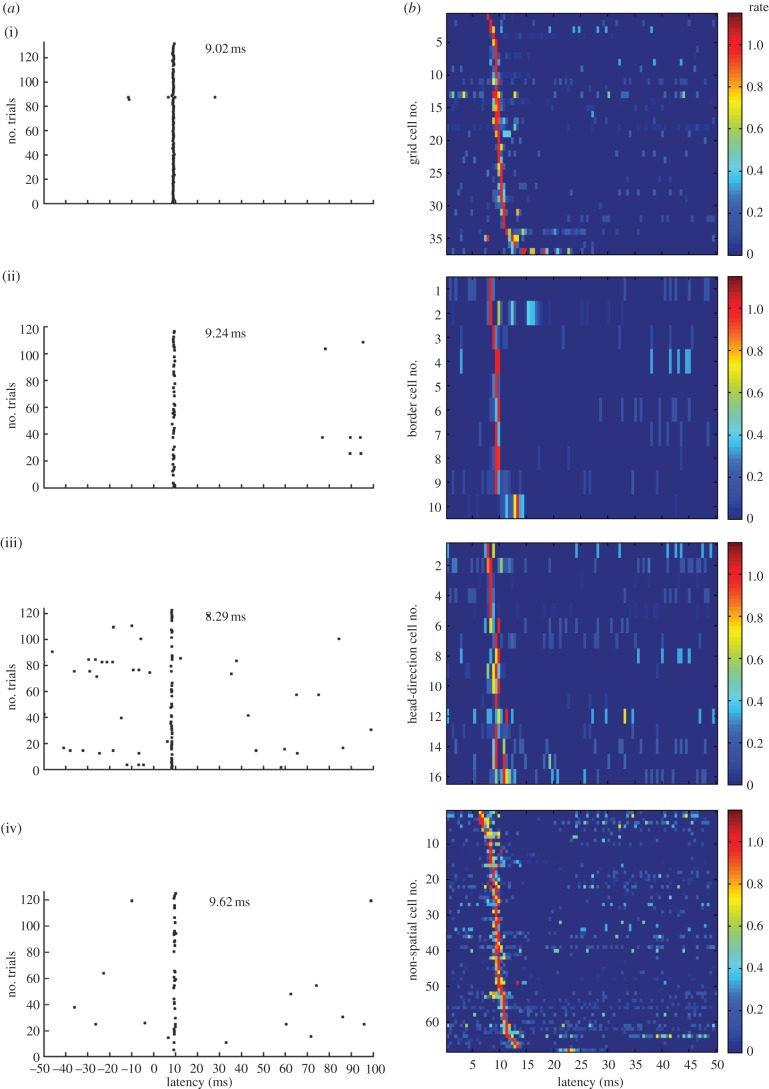
Photoinduced spike activity in multiple functional cell types. (*a*) Spike rasters for the 100 ms following photostimulation above the recording site in the MEC for (i) one grid cell, (ii) one border cell, (iii) one head-direction cell and (iv) one non-spatial cell. Light was on from 0 to 3.5 ms. Dots indicate spike times. Note reliable discharge at a fixed latency after the onset of stimulation. (*b*) Firing latencies for the entire sample of photoresponsive MEC neurons. Colour-coded firing rates are shown as a function of time after the start of photostimulation (0–50 ms). Each row shows data for one cell. Firing rate is shown for successive time bins of 0.5 ms. Rates are normalized to peak rate. Dark blue is silent, red is peak rate; rates are normalized to peak (colour scale to the right). Modified with permission from [27].

Expression of ChR2 in the subset of hippocampus-projecting MEC neurons allowed us to identify hippocampus-projecting entorhinal neurons as those functionally characterized cells that responded instantaneously to short locally delivered light pulses. Because the conductance of native ChR2 channels is small, we used the photocurrent-enhanced gain-of-function mutant H134R [30], whose expression led to reliable photoinduced discharge in a large number of neurons below the light source, in the superficial layers of the dorsal tip of the MEC [27]. Among the light-responsive neurons, approximately 50% had spatial or directional firing correlates (figure 3). Grid cells were the most abundant functional cell type, accounting for approximately one-quarter of the responsive cell population (27%). The sample of light-activated neurons also included a smaller number of border cells (7%) and head-direction cells (12%). The data suggest that the hippocampus receives a broad spectrum of functional inputs from the MEC. These inputs include grid cells and border cells but also a large heterogeneous group of cells with no detectable stable spatial firing correlates (figure 1), whose contributions to hippocampal firing are less well understood. The method does not, in principle, distinguish between neurons that project to the hippocampus and those that pass through or over the structure, for example via the alveus, although most bypassing axons are likely to have collaterals with terminals in the hippocampus itself.

The breadth of responsive cell types does not necessarily imply that projections from all cell types are direct. Light pulses may evoke action potentials either because the cell expresses ChR2 or because ChR2 is expressed in cells with synaptic connections to the recorded cell. It is possible, however, to distinguish direct and indirect firing on the basis of the cell's latency of discharge in response to the light pulse. Direct activation would be expected to discharge cells at minimal latencies and with minimal variation. Indirect or synaptic activation should be slower, and the variation in spike latencies larger, considering that there may be more than one pathway from the ChR2-expressing cell to the recorded cell [31–33]. The data show that the distribution of MEC firing latencies in animals infected with ChR2-carrying rAAV in the hippocampus is sharp, unimodal and symmetric, with a mean of 9–10 ms in each functional cell type and a standard deviation across the entire cell sample of 1–2 ms (figure 3). If a major subset of the cells had been discharged indirectly, then a right-skewed distribution with scattered long-latency values would be expected. The short and symmetric firing latencies of the light-responsive neurons suggest instead that the discharging cells were activated directly. This interpretation is further supported by the fact that spike latencies were substantially longer (between 16 and 30 ms) when MEC cells were activated by photostimulation of presynaptic fibres, in the alveus of the hippocampus, and it is consistent with the finding that backfiring of MEC neurons from the angular bundle or perforant path led to fast discharge, at latencies just slightly longer than those obtained with light pulses in the MEC itself [27]. Synaptically induced activity was occasionally seen also after stimulation in the MEC, expressed either as secondary spikes at longer latencies (fig. S9C and S14 in [27]) or as longer firing latencies for the primary spike (bottom rows in each panel of figure 3 of this paper, as well as in fig. 6D of [27]). However, the incidence of such extended latencies was low, in agreement with the near absence of excitatory connections between stellate cells in the MEC [31–33]. Taken together, these observations imply that, following light pulses in MEC, discharge was normally caused by ChR2 photocurrents in the recorded cell itself.

While the above spike latencies were remarkably constant across cells and animals (figure 3), the range of reported spike latencies varies across studies. In anaesthetized mice, local light flashes led to stereotyped discharges at a mean latency of 9.9 ms in layer V pyramidal cells of the primary motor cortex [34]. This is almost identical to the average latency in our entorhinal study. Photostimulation in ChR2-expressing cultured hippocampal neurons discharged cells slightly faster, at an average latency of 8.0 ms [30]. Similar stimulation of pyramidal neurons in cortical slices led to light-evoked firing at latencies ranging from 3 to 11 ms, with low jitter within cells and large differences between cells [35–37]. The latter datasets were obtained in the presence of glutamatergic blockers, suggesting (i) that the cells were discharged by ChR2 conductances in the recorded cells themselves and (ii) that certain expression patterns can, indeed, lead to long intracellular activation latencies under certain test conditions. The fastest responses have been reported in the somatosensory cortex after expression of ChR2 in fast-spiking interneurons [38], which generally have a lower input resistance, lower capacitance and different dendritic cable properties. The variation in firing latencies across studies may also have a number other causes, including differences in preparation (*in vivo* versus *in vitro* slices or cultured neurons), differences in transgene delivery (*in utero* electroporation versus transfected plasmid, viral transduction or transgenic animals), differences in expression intensity and time and differences in the intensity of the stimulation.

**Figure 4.**
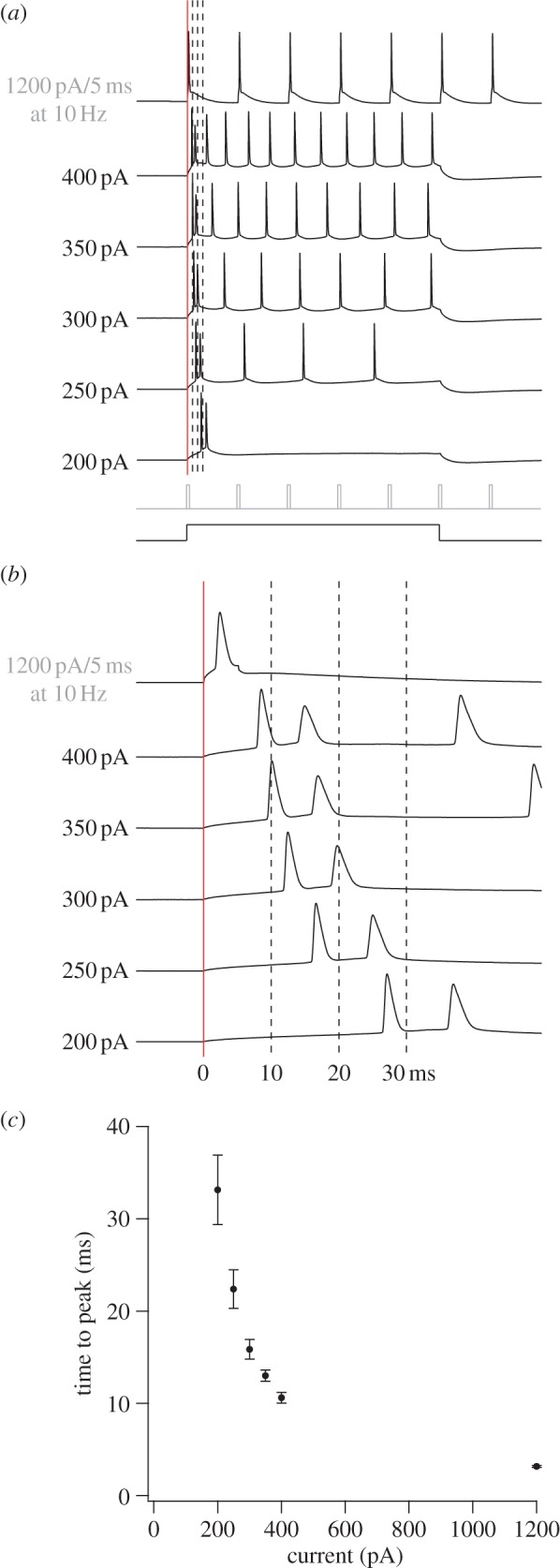
Firing latencies of stellate cells in MEC following intracellular current injection. (*a*) Series of whole-cell current steps in a P42 layer II stellate cell. Schematic of the 500 ms/5 ms current steps is shown below the data (500 ms for 200–400 pA; 5 ms at 10 Hz for 1200 pA). (*b*) Expanded view of 60 ms segment where vertical lines correspond to those also in (*a*). Red line indicates start of current step. (*c*) Graph of mean time to peak values (±s.e.m.) as a function of current, derived from 16 adult stellate cells (P31–P49).

**Figure 5.**
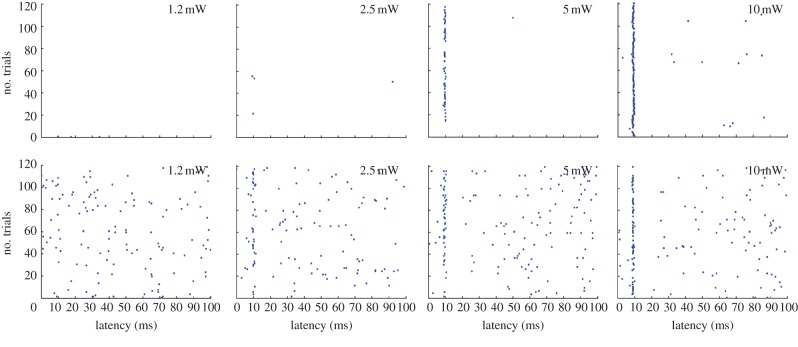
Effect of power density (photostimulation intensity) on response latency for two example cells in MEC (top and bottom rows, respectively). Raster diagrams show light-induced firing at power densities increasing from 1 to 10 mW mm^−2^. Each row shows the first 100 ms of one stimulus period (trial). Dots indicate spike times. Note reliable discharge at an almost fixed latency (approx. 9–10 ms) across a wide range of intensities (2.5–10 mW mm^−2^). Adapted with permission from [27].

It may appear surprising that it took almost 10 ms to discharge a ChR2-expressing principal cell in the *in vivo* studies [27,34]. To test the extent to which late firing reflects low conductances, we applied steps of intracellular current in whole-cell recordings from stellate cells in horizontal MEC slices (figure 4). Current steps between 50 and 400 pA were compared with a command current pulse used specifically to generate precisely timed spike train *in vitro* (1200 pA). The two smallest steps did not reliably elicit spikes. Currents above 200 pA induced firing, with latencies decreasing from more than 30 ms at the lowest intensity to 10 ms at 400 pA and less than 5 ms at 1200 pA. Spike latencies were shorter in recordings from fast-spiking interneurons (3.9 ± 0.3 ms at 400 pA, 10 cells from P21 to P28, mean ± s.e.m.). Standard deviations were extremely small, in accordance with the minimal variation observed in response to light flashes *in vivo*. Taken together, the intracellular current injections suggest that minimal latencies of 9–10 ms are in the range of values to be expected for principal cells in the MEC with low-to-moderate light-evoked conductances. They also show that latencies change only moderately within a rather large range of suprathreshold current amplitudes in the 400 pA region. A perhaps even more striking lack of sensitivity to stimulation intensities was observed in MEC cells *in vivo* (figure 5). In these cells, spike latencies exhibited little variation at power densities above 2.5 mW mm^−2^, which is well below the 10 mW mm^−2^ setting used for identification of hippocampus-projecting cell types. Because of the rather minimal change in spike latency with increasing power density, it is likely that the latencies were also not substantially affected by the distance between the optic fibre and the recorded cells. The reasons for the relative constancy of firing latencies in the *in vivo* preparation remain to be determined, but the *in vitro* study suggests that variations in current amplitude matter less when light intensities are strong, as they were in the *in vivo* study. With sufficiently strong intensities, the large amounts of positive charge that enter the cell may override natural variations in the membrane potential, resulting in relatively constant firing latencies.

## 3. Implications for place cell formation

A role for grid cells in the formation of place cells was suggested as soon as grid cells were identified as a major cell type of the MEC. It was first proposed that localized firing is generated in the hippocampus by linear summation of inputs from grid cells with different grid spacing and grid orientation but overlapping grid phases [39–43]. An alternative possibility was that place cells are generated from more random combinations of inputs from grid cells, in the presence of Hebbian plasticity [44–46] or with the help of local feedback mechanisms [47,48]. The presence of a strong input from grid cells in the optogenetics experiment [27] is consistent with a role for these cells in the formation of place cells. However, the existence of additional inputs, from cells with other spatial correlates or no spatial correlate at all, introduces a need for local mechanisms for the hippocampus to be able to extract or enhance inputs from grid cells and maybe specific subpopulations of grid cells [49].

The nature of the intrahippocampal mechanisms converting grid signals and other signals from the EC to place signals in the hippocampus remains as one of the key questions to be resolved in the years to come. At this time, we do not know whether different place cells receive different types of input from the EC, making some of them more grid-cell-dependent and others more border-cell-dependent, or whether, alternatively, all place cells receive more or less the same mix of inputs, with differentiation taking place subsequently. Such subsequent mechanisms may involve local excitatory–inhibitory circuits, or they may be predominantly intracellular, as they appear to be in some other cortical cell types. In orientation-selective neurons of the visual cortex, for example, a wide range of orientation preferences at the synaptic input level has been reported to be converted into a highly specific output signal [50], and in the auditory cortex, synapses tuned to different tone frequencies are highly interspersed on the same dendrite, at the same time as the output of the cell is tuned to a single frequency range [51]. The mechanisms for converting functionally distributed synaptic inputs to a selective visual or auditory output signal remain to be determined but whatever the mechanism is, it may share critical elements with intradendritic mechanisms for place-cell formation, if such mechanisms exist.

A key finding of the optogenetics experiment is the identification of border cells with direct inputs to the hippocampus. Although the number of border cells is substantially lower than the number of grid cells, these cells may provide information that by summation, and independently of grid cells, results in a localized signal in hippocampal target cells. The presence of a dual spatial input, from grid cells and border cells, is in agreement with early work suggesting that place cells get path integration and landmark-related inputs from distinct sources [52]. It is also interesting to see that a contribution for cortical cells with border-related firing properties was suggested already before the discovery of any functional cell types in the MEC [53]. Specifically, it was proposed that place cells originate from ‘boundary-vector cells’, cells that fire whenever the animal is at a particular distance and direction from an environmental boundary. Summation of inputs from cells with different boundary-vector relations was shown in a computational model to result in place cells that responded in predictable ways to changes in geometric shape [53–55]. The presence of entorhinal border cells with projections to the hippocampus revitalizes these ideas, although it should be noted that whereas the original model generated place fields from cells with firing fields at a continuous range of distances from the walls, nearly all cells in the experimental data from MEC have firing fields that directly touch the peripheral boundaries [25,27]. Whether place cells can be formed only from this subset of the population, and whether the properties of such place cells match those of observed cells, remains to be determined.

The optogenetics data suggest that place fields can be generated in more than one way. In doing so, they may account for the observation that in young rats, place cells appear at a stage of development when the inhibitory network of MEC is not fully matured and grid cells lack the strict periodicity of adult cells [32,56,57]. The data may also account for the finding that place cells can be observed after interventions that disrupt grid-cell firing in the MEC, such as inactivation of the medial septum [58]. In both cases, spatial firing might be maintained by geometrical inputs from border cells. The findings would be consistent with a dual set of spatial inputs to the hippocampus [52], where grid cells provide the place cells with a spatial metric, whereas border cells provide them with information about geometrical relationships [59,60]. If this is true, then the metric properties of place cells [61,62] should be impaired under conditions with low spatial periodicity in entorhinal grid cells. Whether grid cells and border cells contact the same place cells or different subsets, and whether place cells that receive inputs from multiple sources can dynamically amplify inputs of one type or the other, is not known. Addressing the detailed mechanisms of place field formation would require genetic tagging at the level of individual cells—a technology that is on the horizon, after the emergence of single-cell monosynaptic tracing technologies [63] as well as methods for intracellular recording and stimulation in MEC cells of behaving animals [64,65].

## Funding statement

This work was supported by the Kavli Foundation, two Centre of Excellence grants from the Research Council of Norway (Centre for the Biology of Memory and Centre for Neural Computation) and an Advanced Investigator Grant to E.I.M. from the European Research Council (‘CIRCUIT’, grant agreement no. 232608).
